# Prediction of internal carotid artery aneurysm recurrence by pressure difference at the coil mass surface

**DOI:** 10.1007/s00234-020-02553-2

**Published:** 2020-09-14

**Authors:** Takehiro Uno, Kouichi Misaki, Iku Nambu, Akifumi Yoshikawa, Tomoya Kamide, Naoyuki Uchiyama, Mitsutoshi Nakada

**Affiliations:** Department of Neurosurgery, Graduate School of Medical Science, Kanazawa University, 13-1 Takara-machi, Kanazawa, Ishikawa 920-8641 Japan

**Keywords:** Pressure difference, Virtual post-coiling model, Intracranial aneurysm, Computational fluid dynamics, Recurrence after coil embolization

## Abstract

**Purpose:**

A previous study on computational fluid dynamics reported that a high pressure difference (PD) at the surface of a coil mass is a strong predictor of aneurysm recurrence after coil embolization. PD was calculated using a virtual post-coiling model (VM), created by manually cutting the aneurysm by the flat plane from an anatomic model created with pre-coil embolization data; however, its credibility has not been fully evaluated. This study aims to clarify whether PD values calculated using the post-coiling model, which reflects the actual coil plane, are a strong predictor of aneurysm recurrence.

**Methods:**

Fifty internal carotid artery aneurysms treated with endovascular coil embolization were analyzed (7 recanalized, 43 stable). We created and subjected two post-coiling models, namely, VM and the real post-coiling model (RM), constructed from the post-coil embolization data. The relationship between PD and aneurysm recurrence was examined using these models. PD and its constituent three parameters were compared between VM and RM.

**Results:**

PD values calculated using RM showed significantly higher aneurysm recurrence in recurrence group than stable group (*p* < 0.001), and multivariate analysis showed that PD in RM (*p* = 0.02; odds ratio, 36.24) was significantly associated with aneurysm recurrence. The receiver operating characteristic analysis revealed that PD values accurately predicted aneurysm recurrence (area under the curve, 0.977; cutoff value, 3.08; sensitivity, 100%; specificity, 97.7%). All four parameters showed a significant correlation with VM and RM (*p* < 0.001).

**Conclusion:**

Use of PD to predict recurrence after coil embolization can be clinically relevant.

## Introduction

Endovascular coil embolization is a safe and reliable technique for intracranial aneurysm treatment [1, 2]. However, aneurysms are more likely to recur after coil embolization than after surgical clipping [3–6]. It is crucial to investigate the recurrence rate after coil embolization and actively assess it in the field of computational fluid dynamics (CFD) [7, 8]. Recently, Nambu et al. [9] thoroughly investigated the risk factors for aneurysm recurrence after coil embolization by conducting CFD analysis using the virtual post-coiling model (VM). VM was constructed from the pre-coil embolization data and by manually cutting the aneurysm by the flat plane, thus dividing the aneurysm dome and the origin of the branching vessel or parent artery. They concluded that a high pressure difference (PD) was the strongest predictor for aneurysm recurrence when compared with other morphological and hemodynamic parameters. This result is beneficial because it enables predicting aneurysm recurrence before treatment. However, in the VM, part of the aneurysm was artificially deleted, and the surface of the coil mass, defined as the coil plane, was a virtual plane. Furthermore, the virtual coil plane is morphologically different from the actual coil plane in that it is rough; however, the reliability of VM is unknown. It is necessary to examine whether the same tendency can be obtained using the model reflecting the actual coil plane. We constructed a real post-coiling model (RM), reflecting the actual coil plane, from the post-coil embolization data. In this study, we investigated the role of PD in predicting aneurysm recurrence after coil embolization using VM and RM, and we compared the results of both models.

## Methods

### Source of the patient data

Among patients who underwent endovascular coil embolization for internal carotid artery (ICA) aneurysm treatment at our institution, we selected those who underwent time-of-flight magnetic resonance angiography (TOF-MRA) imaging at follow-up. In particular, we focused on patients who have been followed up for over 1 year and whose radiological data quality has been maintained sufficiently for CFD analysis. All patients had a volume embolization ratio (VER), the ratio of the volume of the aneurysm to the volume of the coil, of at least 20%. Finally, 48 patients with 50 ICA aneurysms (32 in the posterior communicating artery, 11 in the paraclinoid ICA, 4 in the ophthalmic artery, and 3 in the anterior choroidal artery) were included in the study. Of the 50 cases, 15 were ruptured, and 35 were unruptured aneurysms. In this study, recanalized aneurysms were defined as cases with an increase in the Raymond–Roy grade or cases within the grading but required retreatment. We then defined stable aneurysm as cases with no change or enlargement within a grade with no retreatment. The institutional review board approved this study, and prior informed consent was obtained from the patients.

### Aneurysm modeling

**Fig. 1:**
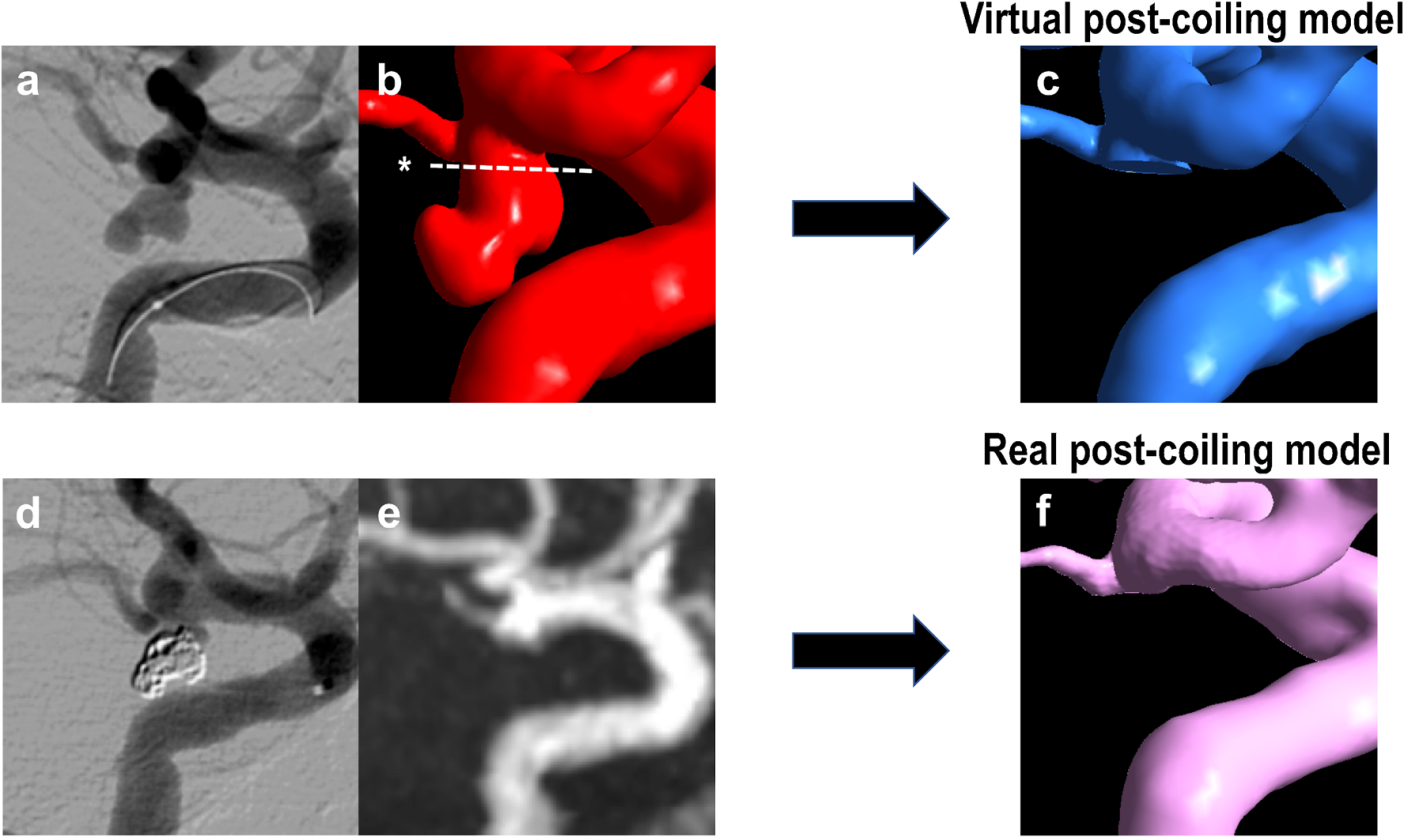
The virtual post-coiling model (VM) constructed using the digital imaging for communication in medicine (DICOM) data of pre-coil embolization three-dimensional rotational angiography (**a**). First, a pre-coiling model is constructed by artificially deleting part of the aneurysm at the coil plane (**b**) (*). The coil plane of VM is flat (**c**). The two-dimensional digital subtraction angiography of the final image after coil embolization is shown (**d**). The real post-coiling model (RM) constructed the DICOM data of post-coil embolization time-of-flight magnetic resonance angiography (**e**). It is created using the data on the actual coil plane obtained from the data after the actual coil embolization. The coil plane of RM is rough and uneven (**f**)

For each aneurysm, both VM and RM were created (Fig. 1). We used the digital imaging for communication in medicine (DICOM) data of three-dimensional rotational angiography (3D-RA) for VM analysis and the TOF-MRA data for RM analysis. TOF-MRA was performed at 1.5 T (SIGNA HDxt; GE Medical Systems, Milwaukee, WI) or 3 T (GE Healthcare, Milwaukee) field strengths. TOF-MRA imaging parameters were as follows: for 1.5 T, 25 ms repetition time (TR), 3.2 ms echo time (TE), 1 number of signal averages (NSA), 1.2 mm section thickness, 200 mm field of view (FOV), and 320 × 192 acquisition matrix, and for 3 T, 24 ms TR, 3.4 ms TE, 0.85 NSA, 1 mm section thickness, 200 mm FOV, and 384 × 224 acquisition matrix. Vascular geometry was extracted using manual cropping and image thresholding. When identifying the boundaries of the geometry of blood vessels from the TOF-MRA and 3D-RA voxel data, the 2-D digital subtraction angiography (DSA) geometry was checked to ensure that the geometry was as close as possible to the reality. Aneurysm and main blood vessels were converted into standard triangulated surfaces using Amira (version 5.6, Maxnet Co. Ltd., Tokyo, Japan). These 3-D images were imported into ICEM CFD software (version 16.2, ANSYS Inc., Canonsburg, Pennsylvania, USA) to construct the aneurysm and blood vessel structures [10]. When creating a blood vessel model, care was taken to maintain the proper length of the mother blood vessel to maintain accurate quality of the numerical simulation.

VM was created using DICOM image data from 3D-RA, which was imaged before coiling. The coil plane in VM was set to a plane where the aneurysm neck and branch blood vessels or parent artery can be separated under the assumption that complete obliteration was achieved. We artificially deleted part of the aneurysm, and the surface had a virtual coil plane, thus creating a VM (Fig. 1a–c). After coiling (Fig. 1d), the RM was created using the DICOM image data from the TOF-MRA images after treatment (Fig. 1e). We used the data that were obtained within a week from coiling to MRA imaging. Unlike VM, where the aneurysm was artificially deleted to create a flat virtual coil plane, RM was created using the actual postoperative DICOM data. The coil plane of the RM was an uneven surface reflecting the actual effect of the coil mass (Fig. 1f). The inlet plane was defined as the section plane of the ICA located 1 mm proximal to the aneurysm, as previously described [10, 11].

### Numerical simulations

We performed CFD simulations as previously described [9, 12, 13]. ANSYS ICEM CFD software was used to create fluid domains for the two vascular models for each patient in order to create meshes with compromised tetrahedrons and seven prism element layers near the wall surface in the boundary. A 75-mm passage was added to the proximal plane of the vessel structure to make the inlet length sufficiently long for simulation [8]. Blood density and dynamic viscosity were defined as 1100 kg/m3 and 0.0036 Pa s, respectively, and were modeled as Newtonian fluids. The condition of the vessel wall was assumed to be a hard slip-free boundary. To solve the pulsatile-flow governing Navier–Stokes equations, we used ANSYS CFX (version 16.2, ANSYS Inc.) [14]. In this study, we used average mass flow rates in the ICA measured from healthy adults, as described by Ford et al., and then a 1.8-s transient analysis was performed [15]. The pressure was set to zero at the outlet, and the same boundary conditions were applied to all models. The calculation interval time was set to 0.005 s, and two cardiac cycles were simulated. The result of the second cycle was used in this study.

### Analysis of hemodynamic parameters in the virtual and real post-coiling models

We examined 4 hemodynamic parameters in both VM and RM. The maximum pressure (Pmax) at the coil plane, average pressure (Pave) at the inlet plane, average velocity (Vin) at the inlet plane, and PD were measured [9]. Pmax, Pave, and Vin are components of PD. In this study, we examined these 4 hemodynamic parameters to investigate the relationship between PD and aneurysm recurrence using VM and RM. Pmax was calculated as the highest value of pressure in the virtual and real coil planes. Pave was calculated as the mean value of the pressure at the inlet plane. Vin was calculated as the average flow velocity at the inlet plane in meters per second. The PD in this study was calculated using the following equation:

$$ \kern0.75em \mathrm{Pressure}\ \mathrm{difference}=\frac{\mathrm{Pmax}-\mathrm{P}\ \mathrm{ave}}{\frac{1}{2}\uprho {Vin}^2} $$

In this equation, *ρ* is 1100 kg/m3. The pressure difference was defined as the degree of pressure elevation at the post-coiling plane from the inlet plane calculated by subtracting Pave from Pmax. This value is normalized by dividing the dynamic pressure at the inlet plane. These values were analyzed at the peak systole.

### Statistical analysis

Continuous data and the number of observations were reported as mean ± standard deviations for continuous variables (frequency, %). Mann-Whitney *U* test or Fisher’s exact test was used to analyze parameters, as appropriate. Hemodynamic parameters were predicted by constructing receiver operating characteristic (ROC) curves and calculating the area under the curve (AUC). Univariate logistic regression model was estimated in order to identify predictive factors of recurrence after coil embolization. Variables statistically significant at univariate analysis were then included in the multivariate logistic regression model to identify independent predictive factors of recurrence after coil embolization. However, Of the values that showed statistically significant different in univariate analysis, PD in VM is a factor similar to PD in RM, and the value of PD in VM, which was considered to be less correlated with recurrence than PD in RM from the ROC curve, was excluded from consideration. Spearman’s rank correlation test was used to determine numeric relationships between the values calculated using VM and those calculated using RM. Statistical significance was indicated when *p* < 0.05. Statistical analysis was performed using SPSS (IBM SPSS Statistics 24, Chicago, IL, USA).

## Results

**Fig. 2:**
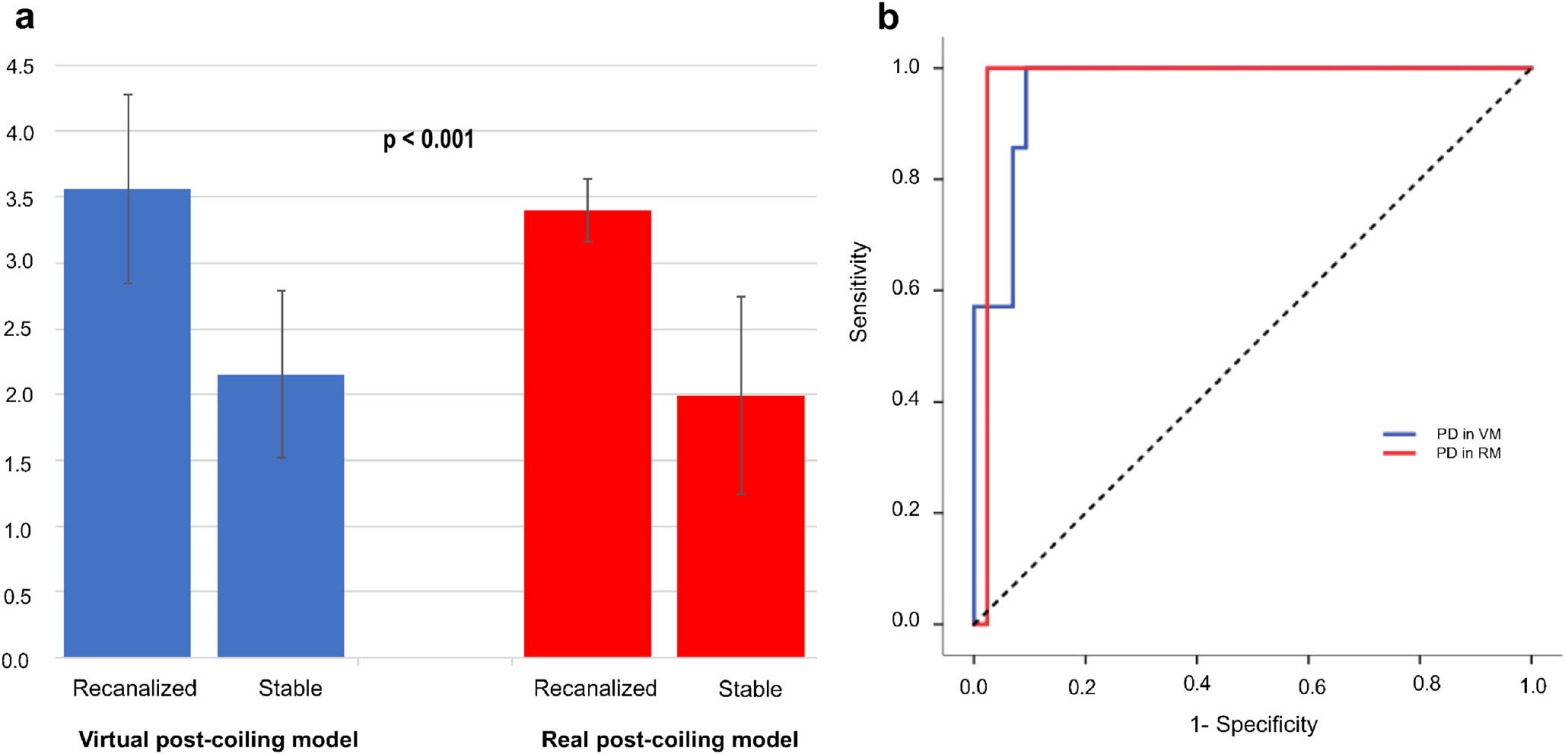
The relationship between pressure difference (PD) and recanalized aneurysm in the virtual post-coiling model and real post-coiling model. **a** Comparison of PD values between the recanalized and stable groups. **b** Receiver operating characteristic curve of PD for predicting recanalization after coil embolization in the coil plane. AUC, area under the curve

**Fig. 3:**
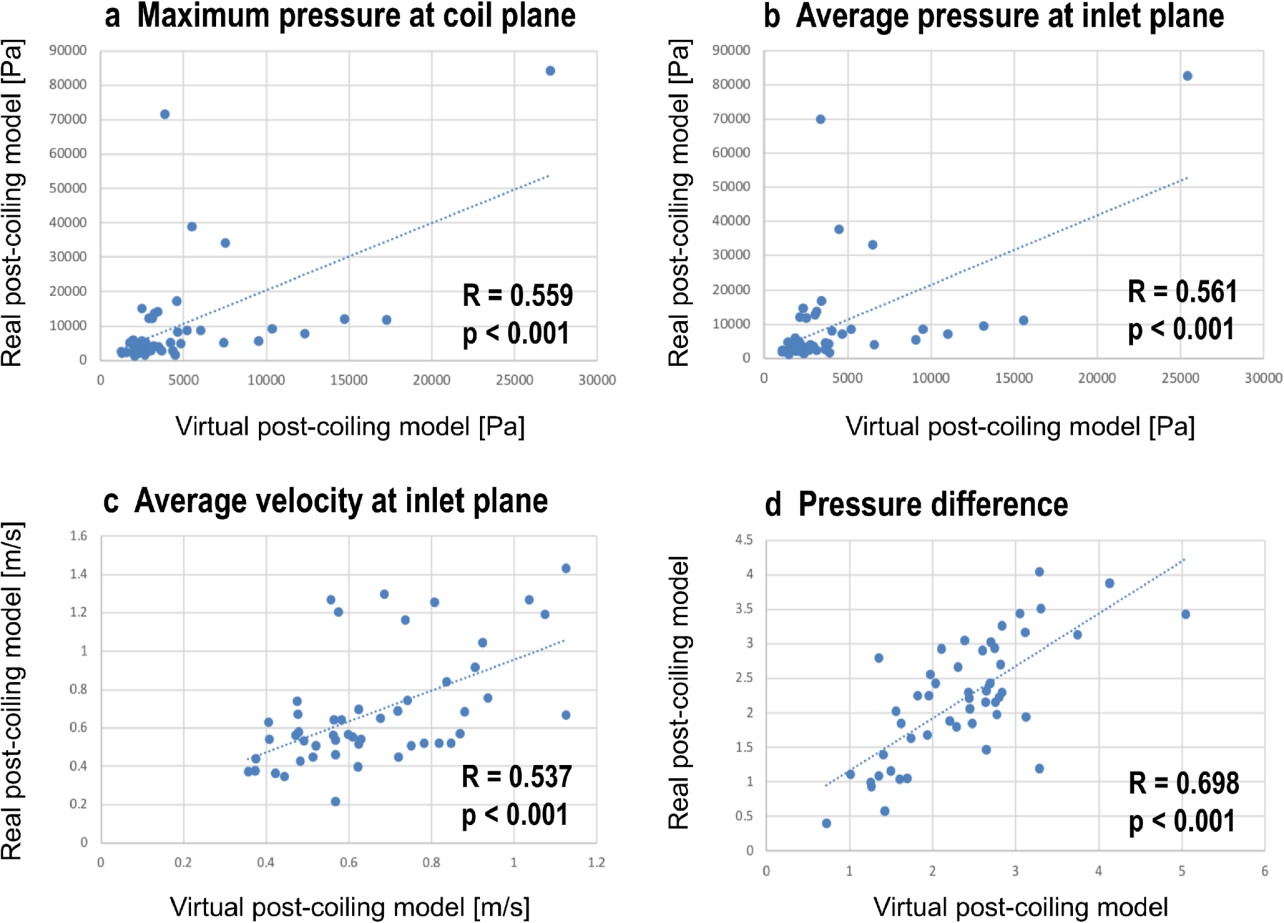
Examples of correlation charts between pressure difference and the three components of pressure difference for virtual post-coiling model and real post-coiling model. **a** Maximum pressure at the coil plane. **b** Average pressure at the inlet plane. **c** Average velocity at the inlet plane. **d** Pressure difference

Seven of the 50 aneurysms (14.0%) were classified into the recanalized aneurysm group. All of them were located at the posterior communicating artery (PcomA). Comparisons of patients’ and morphological and hemodynamic characteristics between the recanalized group and the stable group are shown in Table 1. For morphological features, maximum size, and percentage of ruptured aneurysms were significantly higher in the recanalized group than in the stable group. There was no significant difference in the association of PcomA with fetal type and recurrence. Regarding hemodynamic parameters, no significant differences were found in terms of the three components of PD; however, PD was significantly higher in the recanalized aneurysm group than in the stable aneurysm group. VM and RM showed a similar tendency. Only PD in RM (odds ratio: 36.24, 95% confidence interval 1.47–893.69; *p* = 0.02) showed a statistically significant difference when multivariate logistic regression analysis was performed on values including maximum size, PcomA aneurysm, ruptured aneurysm and PD in RM. When we focused on the value calculated using VM and RM (Fig. 2), PD in VM was 3.60 ± 0.78 in the recurrence group, significantly higher than that of 2.15 ± 0.64 in the stable group (*p* < 0.001), and PD in RM was 3.40 ± 0.24 in the recurrence group, significantly higher than that of 1.99 ± 0.75 in the stable group (*p* < 0.001, Fig. 2a). The ROC analysis for PD in VM and RM revealed an AUC of 0.967, a sensitivity of 100%, and a specificity of 90.7% when the cutoff value was 2.83 in VM and AUC of 0.977, a sensitivity of 100%, and a specificity of 97.7% when the cutoff value was 3.08 in RM, respectively (Fig. 2b). Similar to the VM results, cases with a high PD also recurred after coil embolization in the RM results. PD, Pmax, Pave, and Vin showed significant correlation with VM and RM (*p* < 0.001, Fig. 3). The correlation coefficient was 0.698 for PD, 0.559 for Pmax, 0.561 for Pave, and 0.537 for Vin.

**Table 1:** Patient and morphological and hemodynamic characteristics

	Univariate analysis			Multivariate analysis		
	Recanalized group (*n* = 7)	Stable group (*n* = 43)	*p* value*	OR	95% CI	*p* value
Sex, *n* (%)			0.14		Not evaluated	
Male	2 (28.6)	3 (7.0)			Not evaluated	
Female	5 (71.4)	40 (93.0)			Not evaluated	
Age, years	65 ± 14	58 ± 13	0.20		Not evaluated	
Maximum size, mm	11.5 ± 2.6	8.3 ± 2.5	0.01			0.20
PcomA aneurysm (%)	7 (100)	25 (58.1)	0.04			0.99
Ruptured aneurysm (%)	5 (71.4)	10 (23.3)	0.02			0.07
VER, %	22.5 ± 3.50	24.8 ± 4.5	0.16		Not evaluated	
Pmax in VM, Pa	3815.09 ± 3014.30	4440.71 ± 4597.14	0.89		Not evaluated	
Pave in VM, Pa	3679.95 ± 3031.15	4267.22 ± 4554.09	0.81		Not evaluated	
Vin in VM, m/s	0.63 ± 0.17	0.66 ± 0.20	0.81		Not evaluated	
PD in VM	3.60 ± 0.78	2.15 ± 0.64	< 0.001		Not evaluated	
Pmax in RM, Pa	6236.32 ± 4801.38	10,617.80 ± 16,704.73	0.83		Not evaluated	
Pave in RM, Pa	5672.88 ± 4789.61	10,039.57 ± 16,397.22	0.74		Not evaluated	
Vin in RM, m/s	0.54 ± 0.10	0.70 ± 0.31	0.41		Not evaluated	
PD in RM	3.40 ± 0.24	1.99 ± 0.75	< 0.001	36.24	1.47–893.69	0.02

PcomA, posterior communicating artery; VER, volume embolization ratio; Pmax, maximum pressure at coil plane; Pave, average pressure at inlet plane; Vin, mean velocity of at inlet; PD, pressure difference; VM, virtual post-coiling model; RM, real post-coiling model; OR, odds ratio; 95% CI, 95% confidence interval. Values are shown as mean ± SD when appropriate

*The *t* test and Mann-Whitney *U* test were used; *p* < 0.05 was considered statistically significant

## Illustrative cases

### Recanalized case

**Fig. 4:**
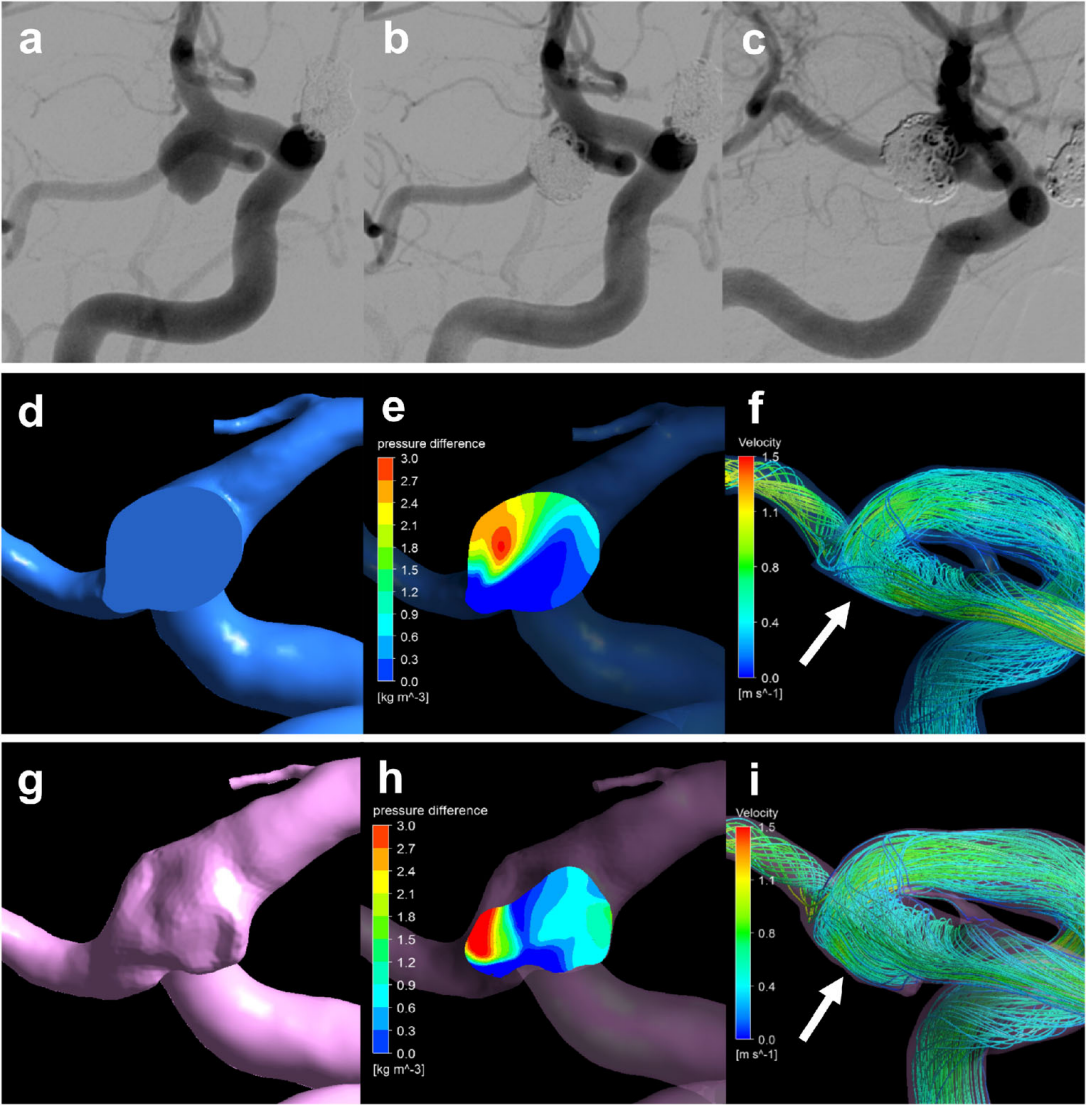
Illustrative recurrence case of a 66-year-old woman with a ruptured PcomA aneurysm. This aneurysm was treated by coil embolization with 20.6% of the final VER and recurrence at 6 months after treatment. **a** Pre-op intraoperative image. **b** Postoperative image. **c** Follow-up image. We created a virtual post-coiling model (VM) (**d**) and a real post-coiling model (RM) (**g**). The contour lines of the pressure difference of VM (**e**) and RM (**h**) are shown in the figures, and the high pressure difference (PD) area is near the branch vessels. The PD values of this case were 3.05 in VM and 3.43 in RM. The streamlines of VM (**f**) and RM (**i**) are shown in the figures, and the white arrow indicates the coil plane. Blood flow collides with the coil plane and branches

A ruptured PcomA aneurysm was found in a 66-year-old woman (Fig. 4). The largest diameter of this aneurysm was 10.8 mm (Fig. 4a). This aneurysm was treated by coil embolization with 20.6% of the final VER (Fig. 4b) and recanalized after 6 months (Fig. 4c). The coil planes of VM and RM are demonstrated in Fig. 4d and g, respectively. The contour lines of the pressure difference are depicted on the coil plane of the VM (Fig. 4e) and RM (Fig. 4h). A high PD area was present near the branch vessels in both models. Streamlines of VM (Fig. 4f) and RM (Fig. 4i) are shown in the figures. Blood flow collided with the coil planes and diverged to the branch vessels and parent arteries in both models. The PD values in this case were 3.05 and 3.43 in VM and RM, respectively.

### Stable case

**Fig. 5:**
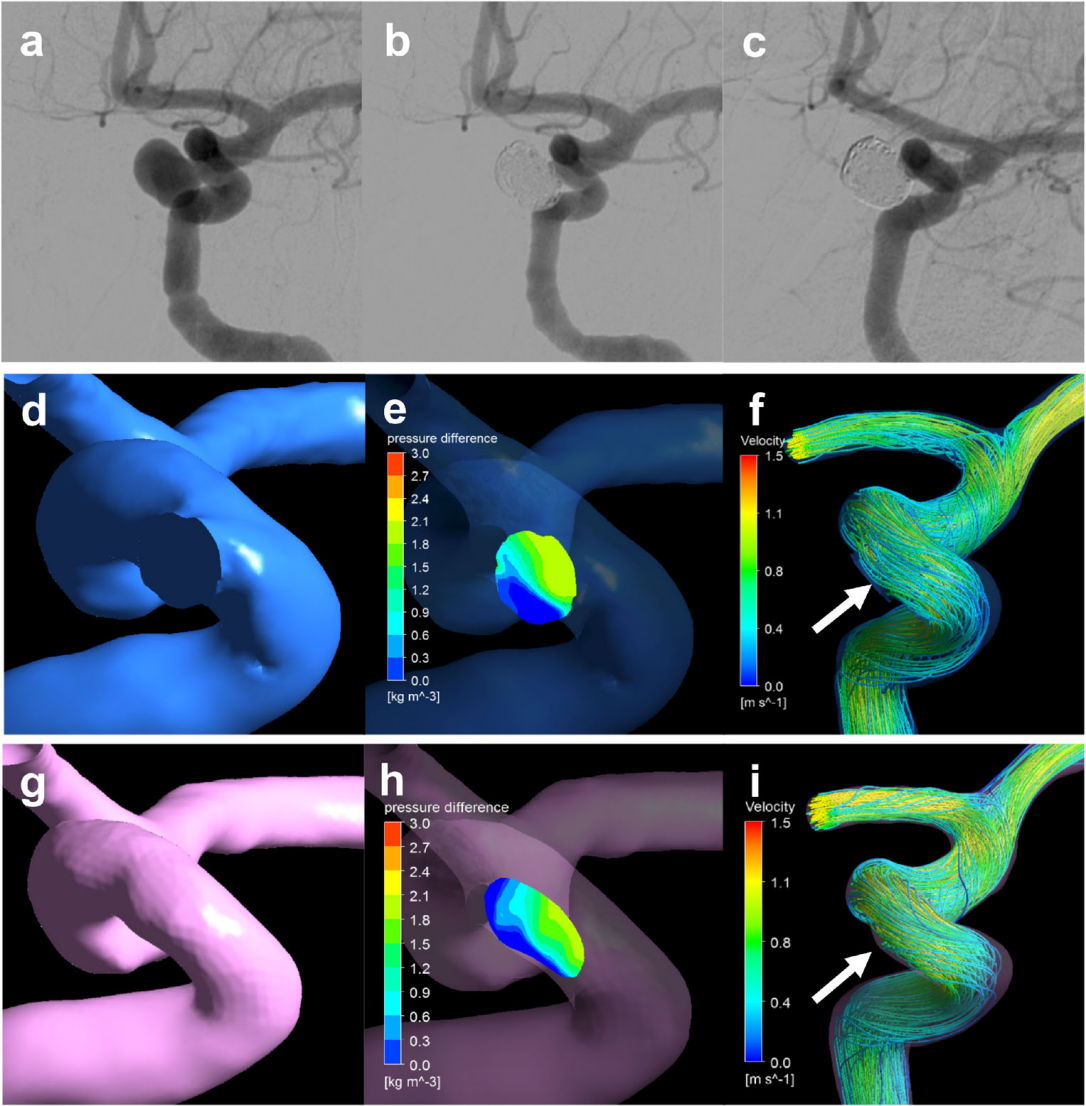
Illustrative stable unruptured paraclinoid aneurysm in a 44-year-old woman. The aneurysm was treated by coil embolization with 23.3% of the final VER. **a** Pre-op intraoperative image. **b** Postoperative image. **c** Follow-up image. We created a virtual post-coiling model (VM) (**d**) and a real post-coiling model (RM) (**g**). The contour lines of the pressure difference of VM (**e**) and RM (**h**) are depicted in the figures, and the high pressure difference (PD) area is proximal to the coil plane. The PD value in this case was 2.65 in VM and 2.80 in RM. The streamlines of VM (**f**) and RM (**i**) are depicted in the figures, and the white arrow indicates the coil plane. Blood flow along the coil plane

An unruptured paraclinoid aneurysm was diagnosed in a 44-year-old man (Fig. 5). The largest diameter of this aneurysm was 9.4 mm (Fig. 5a). This aneurysm was treated using coil embolization with 23.3% of the final VER (Fig. 5b) and did not recanalize at 12 months after treatment (Fig. 5c). The coil planes of VM and RM are shown in Fig. 5d and g. The contour lines of the pressure difference are depicted on the coil plane of VM (Fig. 5e) and RM (Fig. 5h). A high PD area was present on the proximal side of the coil plane. Streamlines of VM (Fig. 5f) and RM (Fig. 5i) are shown in the figures. Blood flow was observed along the coil plane in both models. The PD values of this case were 2.30 and 2.80 in VM and RM, respectively.

## Discussion

We investigated for recurrent factors after coil embolization with RM constructed from the TOF-MRA data after coil embolization. Nambu et al. reported a significantly higher PD value in recurrent cases using VM and an AUC of 0.967, a sensitivity of 100%, and a specificity of 90.7%, when the cutoff value was 2.83 in ROC analysis. The study concluded that among the seven items examined, PD values calculated using VM showed the highest correlation with recurrence and were the strongest predictor of recurrence after coil embolization [9]. PD values calculated using RM were significantly higher in recurrence cases. Additionally, the calculated values using RM showed a significant correlation with VM with respect to PD, and the three parameters were required for the PD calculation. Our results suggest that whether calculated using VM or RM, PD shows a strong association with recurrence and is a useful factor for predicting recurrence.

As for the characteristics of patients in this study, maximum aneurysm size, PcomA aneurysm, and ruptured aneurysm were significantly higher in the recurrence group than in the stable group. Raymond et al. reported that variables that were significant predictors of recurrence included aneurysm size ≥ 10 mm and treatment during the acute phase of rupture [4]. Furthermore, Compi et al. found that coiled aneurysms in the PcomA location had a significantly increased risk of recurrence requiring re-embolization [16]. The characteristics of the patients in this study were similar to those reported previously.

Studies have used VM created by artificially deleting vascular structures from vascular models [9, 17–19]. In these reports, hemodynamic factors such as influence of bleb formation, aneurysm rupture, and recurrence factors after coil embolization were examined. Although VM are models in which structures are artificially deleted, it has not been clarified whether examination in the model reflects an actual in vivo phenomenon. There have been no reports on the verification of VM use for this purpose thus far, and its credibility has not been validated. We investigated the reliability of using VM by comparing the results of VM with those of RM. The results of this study indicate that VM and RM show significant correlation, and these two models are useful for the examination of hemodynamic factors.

Standardizing parameters is a universal index because it standardizes and examines individual differences between each case. A number of studies have examined standardized parameters in the past. PD is a non-dimensionalized value, obtained by dividing the pressure increase from the mother blood vessel to the coil plane by the dynamic pressure. PD can be defined as the degree of pressure increase from the mother blood vessel at the coil plane, which generalizes individual differences among patients [9, 20, 21]. As we adopted the inlet conditions uniformly, parameter values are preferable to be standardized values to account for patient-specific inflow conditions. We speculate that high PD on the coil mass surface may cause coil compaction, resulting in the recurrence of treated aneurysms [22].

PD can be calculated using VM before treatment and is useful for treatment planning. Patients with high PD calculated using VM may need to consider changes in treatment strategy. One method is to drastically change the surgical method from endovascular coil embolization to craniotomy clipping. Clipping is invasive but is known to have a low recurrence rate [3–5]. The recurrence rate would be reduced if patient consent was obtained and clipping treatment was selected. If coil embolization is selected even if the recurrence rate is expected to be high, stent-assisted techniques may be recommended. Previous reports have shown that stent-assisted coil embolization decreases recanalization [23–25]. The underlying mechanism could be as follows: (1) reduced inflow to the aneurysm due to stenting, which promotes thrombus formation; (2) hemodynamic changes due to the straightening effect of the parent artery; and (3) promoting neointimal growth with stents. In cases where high PD as calculated using RM and a high recurrence rate are expected, more frequent follow-ups and additional treatments will be considered, which would lead to a change in treatment policy and would be useful information for the patients. Particularly, in cases of ruptured aneurysms, treatment is often performed as soon as possible after the onset, and it is difficult to calculate PD using VM before treatment. In our results, comparing the results of PD calculated using RM and VM, the results of RM show slightly stronger correlation with aneurysm recurrence than those of VM, and the use of RM is preferred for postoperative follow-up. Furthermore, RM reflects the actual postoperative coil plane, allowing for less biased and objective measurements.

Although clear geometry is essential for CFD analysis, obtaining sufficient quality of the geometry is technically difficult for aneurysms treated with coils. In this study, we used the TOF-MRA data of the actual coil plane after coil embolization to create RM. 3D-RA data are often used for CFD analysis because of the high resolution and accurate vessel visualization. However, TOF-MRA is more often used as a follow-up image than 3D-RA and contrast-enhanced magnetic resonance angiography (CE-MRA) because of its low invasiveness [26, 27]. Ferre et al. reported on the utility of TOF-MRA with DSA in 51 cases and concluded that TOF-MRA was at least as efficient as DSA for the evaluation of intracranial aneurysm occlusion [28]. In addition, van Amerongen et al. performed a systematic review and meta-analysis and reported that TOF-MRA can be used to study recurrence with accuracy comparable with that of DSA. Moreover, they also reported that TOF-MRA is superior to CE-MRA for the follow-up of intracranial aneurysms treatment with endovascular coil occlusion [29]. It is beneficial to use TOF-MRA during the follow-up of recurrence after coiling in clinical practice, and it is also a useful modality to evaluate the coil plane used in a model representing post-coil embolism. There have been several reports on CFD studies on the use of MRA images, and we believe that MRA data are useful for performing CFD analysis [30, 31].

## Limitations

This study has several limitations. First, CFD was performed for only 50 aneurysms in this study. We should perform CFD analysis for many more cases to increase reliability. Second, we evaluated only aneurysms located in the ICA. We should consider those located in other parts such as middle cerebral artery and anterior communicating artery. Third, although it may vary from patient to patient, the boundary condition was uniform in all patients. To analyze the characteristics specific to the patients, there is a method in which boundary conditions are obtained and analyzed using phase-contrast magnetic resonance imaging and transcranial Doppler ultrasonic examination. Fourth, the RM construction was examined using the most recent MRA data after coil embolization, but the degree of thrombus formation in the aneurysm may differ depending on the duration until imaging. The effect of thrombosis may need to be taken into account when considering the coil plane that actually causes recurrence. Fifth, TOF-MRA has been used as the first-line modality in the current follow-up, and there has been no issues regarding quality; however, evaluation with a more accurate modality is required. To express a more accurate coil plane, it is necessary to consider data from modalities generating fewer artifacts, such as silent MRA [32]. Sixth, although PD in RM correlates with PD in VM, *R* = 0.698 is not a strong correlation. PD in RM and PD in VM are hemodynamic parameters calculated from different modalities data and need to be thoroughly examined with respect to their correlation [33]. Further study is needed to reveal which is close to ground truth values of PD. Finally, this was a retrospective study; however, to confirm the relationship between PD and recurrence, prospective studies such as interventions in patients with high preoperative PD detection are required.

## Conclusions

The CFD analysis data obtained using RM showed correlation with those obtained using VM. The analysis data in RM also showed that a high PD at the coil plane was strongly associated with recurrence, similar to that calculated using VM. PD can be considered clinically useful to predict recurrence after coil embolization without the need for invasion in patients.

## Abbreviations

3D-RA: Three-dimensional rotational angiography
AUC: Area under the curve
CE-MRA: Contrast-enhanced magnetic resonance angiography
CFD: Computational fluid dynamics
DICOM: Digital imaging for communication in medicine
DSA: Digital subtraction angiography
ICA: Internal carotid artery
PcomA: Posterior communicating artery
PD: Pressure difference
RM: Real post-coiling model
ROC: Receiver operating characteristic
TOF-MRA: Time-of-flight magnetic resonance angiography
VER: Volume embolization ratio
VM: Virtual post-coiling model
